# Electrocauterization versus Ligation of Lymphatic Vessels to Prevent Lymphocele Development after Kidney Transplantation—A Meta-Analysis

**DOI:** 10.3390/jpm14030256

**Published:** 2024-02-28

**Authors:** Ludwig Matrisch, Hryhoriy Lapshyn, Martin Nitschke, Yannick Rau

**Affiliations:** 1 Department of Internal Medicine I, University Medical Center Schleswig-Holstein, Campus Lübeck, Ratzeburger Allee 160, 23538 Lübeck, Germany; martin.nitschke@uksh.de; 2 Department of Surgery, University Medical Center Schleswig-Holstein, Campus Lübeck, Ratzeburger Allee 160, 23538 Lübeck, Germany; hryhoriy.lapshyn@uksh.de; 3 Transplant Center, University Medical Center Schleswig-Holstein, Campus Lübeck, Ratzeburger Allee 160, 23538 Lübeck, Germany; 4 General Practice Teetzmann, 23879 Mölln, Germany; yannick.rau@gmail.com

**Keywords:** lymphocele, renal transplantation, kidney transplantation, ligation, electrocauterization

## Abstract

**Background:** Lymphoceles are amongst the most common complications following kidney transplantation. Therefore, effective strategies to prevent their development are needed. The ligation of lymphatic vessels has proven to be a successful concept for that purpose. However, whether electrocauterization or suture ligation is more effective is unclear. **Methods:** We conducted a meta-analysis using a random effects model with the log risk ratio as the primary outcome measure. Additionally, an analysis using a random effects model with the raw mean difference in lymphatic sealing time between suture ligation and electrocauterization was performed. Adequate studies were found in a literature search conducted in PubMed, CENTRAL and Web of Science as well as from independent sources. **Results:** A total of 8 studies including 601 patients were included in the analysis. The estimated average log risk ratio based on the random effects model was µ = −0.374 (95% CI: −0.949 to 0.201), which did not differ significantly from zero (z = −1.28, *p* = 0.2). The lymphatic sealing time was 7.28 (95% CI:1.25–13.3) minutes shorter in the electrocauterization group. **Conclusions:** We conclude that neither technique is superior for the purpose of lymphocele prevention post kidney transplantation, and secondary criteria like time savings, cost and surgeons’ preference should be considered in the decision for an optimal outcome.

## 1. Introduction

Kidney transplantation is the preferred treatment for end-stage kidney disease, offering improved quality of life and survival compared to dialysis [1]. However, despite advances in surgical techniques and immunosuppression, postoperative complications remain a significant challenge for surgeons, physicians and patients around the world. One such complication is the development of lymphoceles. These fluid-filled cavities can cause pain, discomfort and even transplant malfunction or graft loss through the application of pressure on the graft as well as its surrounding structures [2]. This is particularly important in the first few months after transplantation, when lymphoceles are most commonly found [3].The overall incidence of lymphoceles after kidney transplantation varies. Up to 33.9% of patients have been reported to develop lymphoceles post transplantation [4]. In 23.6% of patients developing lymphoceles, medical or surgical interventions, including but not limited to ultrasound guided aspiration, drainage with or without sclerotherapy and fenestration of the lymphocele into the peritoneal cavity, are required [5,6]. As such, there is a need to identify effective methods for the prevention of the development of lymphoceles post kidney transplantation. 

Lymphoceles are defined as cavities filled with lymphatic fluid. Their formation after kidney transplantation is thought to be due to the leakage of lymphatic fluid from disrupted lymphatic vessels during surgery. Specifically for kidney transplantation, the transection of lymphatic vessels as part of the dissection of the iliac vasculature plays a major role. The details of the pathophysiology and thereby risk factors associated with the development of lymphoceles are currently the subject of scientific discussion. However, it is clear that surgical techniques influence their postoperative incidence [4]. Serirodom et al. have shown that surgical ligation of the lymphatic vessels during transplantation results in significantly reduced incidences of lymphoceles when compared to performing no specific preventive measures [7].

Different ligation techniques are used by surgeons for the closure of various types of vessels. A common and long-established technique is ligation via suture. Surgeons will first clamp the vessel in question perpendicular to its axis. Most commonly, they will then tie a suture around it before dividing the structure and releasing the clamp. This ensures the reliable occlusion of the vessel. In the case of a lymphatic vessel, the vessel’s stump may also be clamped and then tied down via suture after it has already been cut open. As no significant loss of blood is expected and as lymphatic vessels are not as easy to identify as blood-carrying vessels, the technique is still suitable in this case. Whatever the case, this procedure entails a significant cost of material as well as the temporal extension of the overall procedure. Due to time constraints as well as increased economization, researchers and surgeons alike have looked into alternative methods of vessel closure. One such method is electrocauterization, which has found continuously increasing application since its first implementation in surgical practice. Surgeons use the heat-generating properties of a current to damage vessels and coagulate their tissue and contents until they are properly sealed. This procedure has been established in many fields of surgery and has proved to be a pragmatic alternative to suture ligation in many applications [8]. It is generally considered a faster alternative to suture ligature techniques [9].

Despite the potential benefits of these techniques, there is currently no consensus on their use on lymphatic vessels and their effectiveness in reducing the risk of lymphocele development following kidney transplantation. This leads to uncertainty among surgeons as to whether to invest additional operating time into a subjectively safer sealing method. The aim of this meta-analysis is to determine if this additional use of resources is warranted by current evidence. 

## 2. Materials and Methods

### 2.1. Data Collection

To conduct this meta-analysis, we performed a structured literature search in July of 2023 in PubMed and Web of Science as well as the CENTRAL database to identify relevant studies. In PubMed, we used mesh terms for our research and searched for ligation, kidney transplantation and lymphocele with the search query “(TS = (ligation)) AND TS = (kidney transplantation OR renal transplantation)) AND TS = (lymphocele)”. This culminated in twenty-five results, of which five research articles were found to be suitable for this study. In Web of Science, we used the search query “((ligation) AND (kidney transplantation)) AND (lymphocele)”, culminating in 13 results with no new articles found. In CENTRAL, we used the terms “Kidney transplantation” AND lymphocele”.

**Figure 1:**
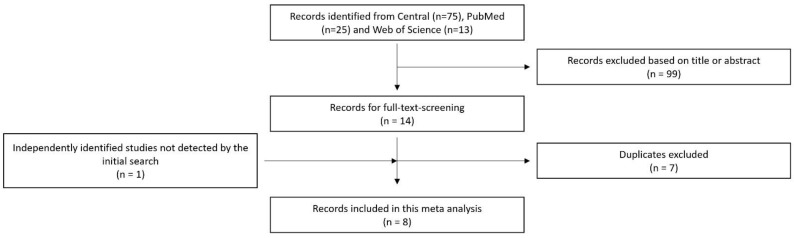
Flow chart representing the data collection process.

After screening the titles and abstracts of the identified studies, we selected those that met our inclusion criteria. These criteria included studies that reported on the use of cauterization or ligation during kidney transplantation and the subsequent development of lymphoceles. We excluded studies that did not report on lymphocele development or that used other surgical techniques for their prevention. Additionally, during our research process, we found one study that was not listed in PubMed, CENTRAL or in Web of Science but was suitable for our purpose by searching for “conventional ligation post-renal transplant lymphoceles” on Google. Furthermore, one suitable study listed in PubMed that was not detectable by our search terms was identified and deemed befitting of the inclusion criteria. This inclusion and exclusion process is further illustrated in Figure 1.

We then extracted data from the included studies on patients’ demographic characteristics, surgical techniques and outcomes. For each study, we recorded the number of patients who developed lymphoceles and divided them into cauterization and suture ligation technique groups. We also collected information on the average age of the patients as well as on lymphatic sealing time (LST) and the studies’ follow up concepts.

To further evaluate the underlying studies, we decided to perform an established quality assessment. Randomized controlled studies were considered of the highest quality. A quality assessment of comparative non-randomized studies was performed using the Newcastle–Ottawa Assessment Scale. A maximum of 9 stars can be achieved by any study subject to the scale [10]. However, there is no definitively defined threshold for what constitutes a high-quality study. Other meta-analyses assume studies with 6 stars or more to be of high quality and those with three to five stars of fair quality, an approach which we adopted for this analysis as well [11,12]. The overall quality of a meta-analysis is significantly influenced by its underlying contents and their quality. An abundance of comparative non-randomized studies could therefore be considered a lack of significant substance and generalizability.

### 2.2. Statistical Analysis

The statistical analysis was carried out using jamovi (Version 2.2.5, The jamovi project, Sydney, Australia), an open-source statistical research tool based on the programming language R which grants a high degree of reproducibility and accessibility. For the analysis of lymphocele development, the log risk ratio was used as the primary outcome measure. The raw mean difference was used as the outcome measure for the analysis of LST discrepancies. We conducted the analysis using a random effects model with the DerSimonian–Laird estimator to estimate the amount of heterogeneity (i.e., tau²) [13]. The Q-test for heterogeneity and the I² statistic are also reported. In the case that any amount of heterogeneity was detected (i.e., tau² > 0, regardless of the results of the Q-test), a prediction interval for the true outcomes is also provided. Studentized residuals and Cook’s distances were used to examine whether studies might be outliers and/or influential in the context of the model. Studies with a Studentized residual larger than the 100 × (1 − 0.05/(2 × k))th percentile of a standard normal distribution were considered potential outliers (i.e., using a Bonferroni correction with two-sided alpha = 0.05 for k studies included in the meta-analysis). Studies with a Cook’s distance larger than the median plus six times the interquartile range of the Cook’s distances were considered to be influential. The rank correlation test and the regression test, using the standard error of the observed outcomes as a predictor, were used to check for funnel plot asymmetry. A Forest plot is provided for each for both models. We also conducted a rank correlation test for funnel plot asymmetry and reported Kendall’s Tau. The results of a regression test for funnel plot asymmetry are provided as well.

## 3. Results

Table 1 presents the key features of the studies included in this meta-analysis. All of the studies mentioned were published in 2017 or later. Three are based on a retrospective study design, and five are prospective randomized controlled trials. Each of them was conducted in a single transplant center. All of them included continuous kidney transplantation patients; however, three patients were excluded based on complications and/or risk factors. Lymphoceles were detected via an ultrasound exam in all of the studies; however, there was no general consensus on the definition of a lymphocele. The follow-up concepts also varied between the trials. In total, 601 patients were included. The average patient was 38.87 years old at the time of transplantation.

### 3.1. Lymphocele Incidence

Out of the 601 patients with kidney transplantations included in this analysis, vessel closure was performed by electrocauterization in 289 (48.1%) cases and by suture ligation in 312 (51.9%) cases. Sixteen (5.5%) lymphoceles were observed in the electrocauterization group and twenty-nine (9.3%) in the suture ligation group, resulting in an overall incidence of forty-five (7.5%) in the total population.

We examined heterogeneity statistics using the DerSimonian–Laird estimator. According to the Q-test, there was no significant amount of heterogeneity in the true outcomes (Q(7) = 4.88, *p* = 0.67, tau² = 0.00, I² = 0.00%).

An examination of the Studentized residuals revealed that none of the studies had a value larger than ±2.73; hence, there was no indication of outliers in the context of this model. According to the Cook’s distances, none of the studies could be considered overly influential. 

Table 2 displays the estimates of the random effects model for lymphocele incidence. A total of k = 8 studies were included in the analysis. The estimated average log risk ratio based on the random effects model was µ = −0.374 (95% CI: −0.949 to 0.201). Therefore, the average outcome did not differ significantly from zero (z = −1.28, *p* = 0.2).

**Figure 2:**
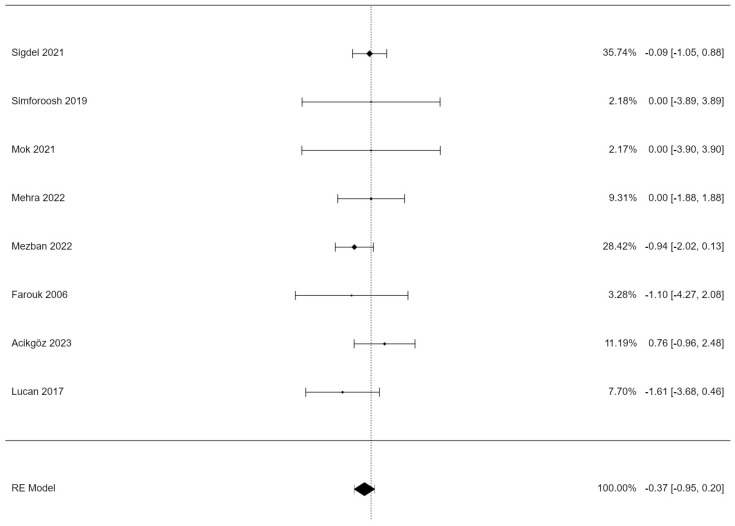
Forest plot of the random effects model. Abbreviations: RE = random effect [14,15,16,17,18,19,20,21].

Figure 2 displays a forest plot of the random effects model. The observed log risk ratios ranged from −1.609 to 0.758, with four out of eight estimates being negative (50%). Three studies reported equal numbers of lymphoceles for both techniques, and Acikgöz et al. reported a higher risk for postoperative lymphoceles after electrocauterization. 

### 3.2. Publication Bias Assessment

**Figure 3:**
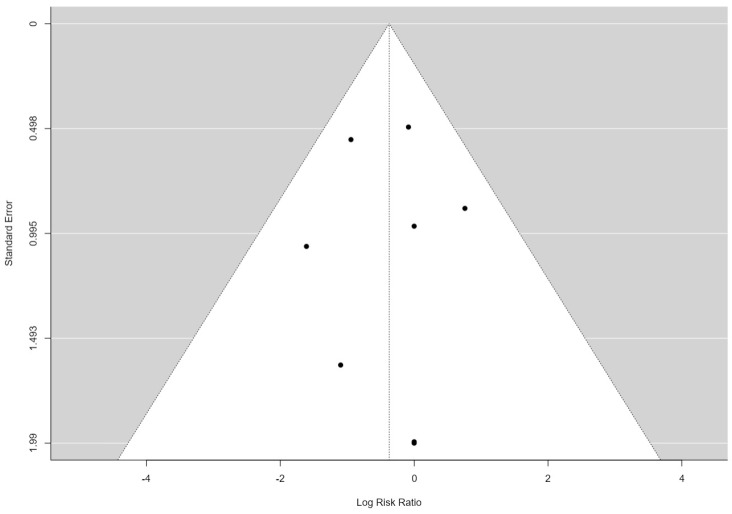
Funnel plot of lymphocele risk. Each dot represents a study analyzed in this meta-analysis.

Figure 3 displays a funnel plot of our analysis. The studies analyzed here are arranged symmetrically, as is also underscored by the rank correlation test for funnel plot asymmetry (Kendall’s Tau = −0.143 and *p* = 0.72) and Egger’s test for funnel plot asymmetry (Z = −0.006 and *p* = 0.995). This suggests a low probability of publication bias. 

### 3.3. Lymphatic Sealing Time

Three studies reported on the lymphatic sealing time (LST) and differences in it between different sealing methods and were included in the following analyses. Overall, the three studies included 175 patients with a mean age of 38.59 years. 

We again examined heterogeneity statistics using the DerSimonian–Laird estimator for the LST. According to the Q-test, there was no significant amount of heterogeneity in the true outcomes (Q(2) = 148.361, *p* < 0.001, tau² = 27.881, I² = 0.00%). A 95% prediction interval for the true outcomes is given by −19.25 to 4.696. Therefore, although the average outcome is estimated to be negative, in some studies, the true outcome may in fact be positive. An examination of the Studentized residuals revealed that one study (Acikgöz 2023) had a value larger than ±2.3940 and may be a potential outlier in the context of this model [21]. According to the Cook’s distances, none of the studies could be considered overly influential. Neither the rank correlation nor the regression test indicated any funnel plot asymmetry (*p* = 1.0000 and *p* = 0.5237, respectively). Overall, our heterogeneity analysis suggests systematic differences between the studies, most likely with regards to how the procedure of sealing was performed in each population. Table 3 displays the results of a random effects model.

**Figure 4:**
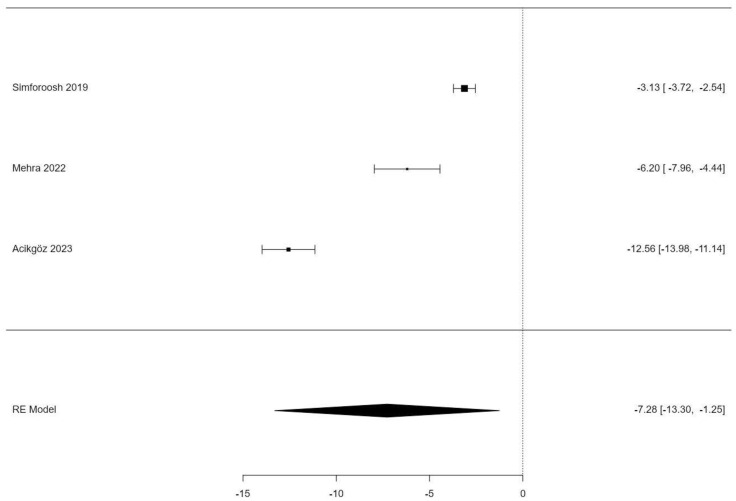
Forest plot of the lymphatic sealing time (LST). Abbreviations: RE = random effect [16,19,21].

Figure 4 shows a forest plot of the studies that reported the lymphatic sealing time. A clear trend of a shortened time frame with LST by electrocauterization can be identified, with all three studies reporting significantly shorter periods. 

## 4. Discussion

To the best of our knowledge, this is the first meta-analysis to discuss the optimal surgical technique for the prevention of lymphocele development post kidney transplantation. This is an important topic in transplantation as lymphoceles are among the more common complications among kidney transplantations. Lymphoceles are not only a common but also a feared complication as they do not only cause pain and discomfort to patients but also carry the risk of transplant failure. Due to the small sample sizes of previous clinical studies and the relevance of the issue for the clinical practice, a meta-analysis was urgently needed.

The issue of the optimal surgical technique to prevent lymphoceles has only come up in clinical research in recent years. This is underscored by the recency of the studies we identified. The first study we analyzed was published in 2019 by Simforoosh et al [16]. This was a surprise since lymphoceles have always been an issue in kidney transplantation [22]. It is not quite clear what sparked the recent scientific interest in the prevention of lymphoceles. 

The available studies were similar in terms of the interventions performed for lymphatic vessel closure as they all used either suture or electrocauterization techniques to ligate lymphatic vessels as preventative measures. However, the follow-up concepts differ between the studies. While Mezban et al., for example, performed weekly ultrasound exams on patients during the first six weeks, Simforoosh et al. only performed one exam to check for lymphoceles five months after surgery. Since the risk of lymphocele formation is considered to be the greatest in the first six weeks, we believe this might play an important role in the accurate detection of lymphoceles and might therefore significantly influence the reported incidences [3]. To account for this, we report the results of a random effects model. In our analysis of the available literature, we found no significant differences between the two techniques. We therefore infer that from an outcome-oriented standpoint, there is no preferable mode of lymphatic vessel closure in kidney transplantation between the two. This opens room for discussion regarding secondary criteria in the decisions of surgeons between these two methods of vessel closure. One such criterion could be the LST, i.e., the time spent on lymphatic vessel closure during surgery. Out of the six studies, two reported the operating time and three reported the LST. We conducted a second analysis to examine the difference in mean LST values between electrocauterization and ligature sealing. The LST was 7.28 (95% CI:1.25–13.3) minutes shorter in the electrocauterization group (*p* = 0.018). Our analysis of heterogeneity regarding the LST suggests that the population within Acikgöz’s study in particular underwent a systematically different sealing procedure when it comes to time spent. This may be due to differences in how meticulously each surgeon performed lymphatic sealing overall. It can also be shown that a shorter LST also correlates with a shorter surgery time. 

This potentially entails tremendous benefits for patients as risks associated with longer procedure times may be reduced by decreasing the LST and therefore the operating time. One analysis of 1564 patients who underwent kidney transplantation, for example, shows that the duration of surgery was an independent risk factor for the development of incisional hernias [23]. Also, an elongated surgery time carries a greater risk of surgical site infections in kidney transplantation [24]. Since kidney-transplanted patients are immunosuppressed due to their medication, infections are dreaded complications. This should be considered in the choice of lymphatic vessel closure technique.

While the presented time frame may not be clinically significant in most cases, economic considerations need to be addressed as well. Previous authors estimated a minute in the operating theater to cost around USD 36 to 46, accumulating a relevant amount when considering budgetary restraints as a common problem in healthcare systems around the world [25,26]. This, however, does not yet include material costs for the ligation itself. This cost heavily depends on the used materials and suppliers and many more factors that cannot be easily estimated. Although, it is reasonable to assume that cauterization devices that may be reused are cheaper than the utilization of single-use sutures, this train of thought becomes somewhat mute when considering that modern surgery almost always requires the availability of an electrocauterization tool at some point (wound closure, bleeding reduction, etc.); thus, such a device will virtually always be used within the procedure, no matter the preferred ligation technique. 

Considering the similarities between the prevention of lymphoceles in patients who undergo kidney transplantation surgery and patients who undergo other procedures involving the lymphatic system, other analyses with similar topics are appropriate. A previous meta-analysis of electrocauterization during axillary lymphadenectomy demonstrated it to be an equally safe option when compared to conventional suture ligation but also showed an increased incidence of seroma formation. This suggests a dependence on anatomical locality in regards to postoperative lymphatic complications [27]. 

The considerations presented above are potentially integral to the decision-making process of surgeons regarding the implementation of different techniques. Personalized approaches are becoming the norm of modern medical care. This also includes controversial topics like the ligation of lymphatic vessels. The creation and review of available evidence like this meta-analysis enables physicians and surgeons to base their individualized therapeutic decisions on facts rather than opinion.

### Limitations

The generalizability of this study’s results is impaired due to a number of factors. Firstly, there were only a few studies available for the investigation of our research question. Also, in these studies, the general incidence of lymphoceles was quite low. Therefore, so is the statistical power. The low incidence can be attributed to a lack of rigorous follow-up concepts for the screening of lymphoceles in most of the studies included. A statistically significant and clinically relevant difference might become apparent in larger patient collectives and/or closer monitoring for lymphocele development. As a result, more research in larger cohorts with more sophisticated study designs is needed to tackle these issues. Also, most of the studies ever published on this issue were published by Asian working groups conducting their research on Asian patients in Asia. This impairs the generalizability of the results. Differences in country-specific health systems might have influenced the results. Moreover, as the pathophysiology of lymphoceles is not fully understood yet, a genetic influence cannot be ruled out, and therefore, genetic similarities in adjacent geographic regions should be taken into consideration. Similarly, this applies to environmental influences. Generalizability is also impaired since not all studies used the same electrocauterization system. Although the underlying mechanism of various electrocauterization devices is the same, technical differences might result in different outcomes in terms of lymphocele development as well as the LST. The same issue may also be brought up with the sutures employed. While self-resorbing sutures are the standard for ligation, suture strength and materials may vary not only from operating theater to operating theater but also from surgeon to surgeon. As the employed materials were not consistently disclosed or described by the authors of the underlying studies, we were unable to analyze a potential impact. The average age of patients, 38.87 years, is lower than what would be expected in transplant centers in more developed regions of the world, also reducing generalizability [28]. Considering the statistical analysis, the use of the estimator of DerSimonian and Laird may also impact this study’s statistical properties in a negative manner as it was shown by other researchers to be prone to issues if only few studies are included within an analysis [29]. Our analysis of the three included retrospective cohort studies showed a range from fair- to high-quality studies that we assessed to be of suitable caliber for this meta-analysis. Overall, we consider the inclusion of three non-randomized trials to not be a significant decrease in the meaningfulness of our analysis as the overall availability of studies regarding the topic in question is scarce. It needs to be noted that a meta-analysis consisting solely of randomized controlled trials would be considered of higher value. However, the lack of information regarding details on the follow-up of patients included within Acikgöz’s study especially limits this study’s value within the overall context of this analysis.

## 5. Conclusions

Until now, it has been unclear whether electrocauterization or suture ligation is the superior choice to prevent lymphoceles after kidney transplantation. We carried out a meta-analysis of all the available trials that looked at this question. We conclude that neither technique is superior for this purpose, and secondary criteria like time savings, cost savings and surgeons’ preference should be considered in the decision for an optimal outcome. These criteria favor electrocauterization. However, further research in larger and more diverse patient populations is needed to increase the generalizability of the results. However, our findings do enable surgeons to better justify individualized approaches in personalized surgical procedures. 

## Figures and Tables

**Table 1:** Studies included in the analysis.

Name	Study Type	Newcastle–Ottawa Scale	Exclusion Criteria	Lymphocele Definition	Follow-Up Regime	Study Size	Average Age in Years (SD)	Duration (in Minutes) Comparison (SD)
Farouk, 2006 [14]	Prospective RCT	-	None	Not specified	Repeated ultrasound exams in the first six months	90	38 (NA)	OT: 95.98 (10.51) vs. 109.78 (12.24)
Lucan, 2017 [15]	Prospective RCT	-	None	Not specified	Not specified	48	40.96 (12.81)	Not provided
Simforoosh, 2019 [16]	Prospective RCT	-	None	Not specified	Once at 5 months	60	41.25 (15.98)	LST: 3.57 (1.09) vs. 6.7 (1.22)
Mok, 2021 [17]	Retrospective cohort study	7 *	None	Not specified	Ultrasound after 1 month, check for symptoms for 2 more months	100	47.44 (12.4)	OT:173.38(29.55) vs. 183(37.14)
Sigdel, 2021 [18]	Prospective RCT	-	Urinary leakage, urinoma formation, retroperitoneal hemorrhage, re-exploration or death within 3 months	>50 mL	Ultrasound after 6 weeks and after 3 months	58	33.6 (10.65)	Not provided
Mehra, 2022 [19]	Prospective RCT	-	Not specified	Not specified	Not specified	52	35.9 (13.75)	LST: 8.3 (1.9) vs. 14.5 (4)
Mezban, 2022 [20]	Retrospective cohort study	6 *	Uncontrolled diabetes, high doses of immunosuppressive drugs, diuretics treatments and anti-coagulants and BMI > 24 or age >60 years	Not specified	Weekly ultrasound for 6 weeks	130	34.22 (10.31)	OT: 140.33 (17.07) vs. 155.57 (17.9)
Acikgöz, 2023 [21]	Retrospective cohort study	5 *	Deceased donor re-transplantation	>50 mL	Not specified	63	38.29 (12.47)	LST: 7.85 (1.97) vs. 20.41 (3.62)

Abbreviations: RCT = randomized controlled trial; SD = standard deviation; LST = lymphatic sealing time; OT = operation time; BMI = body mass index; mL = milliliter. In the column “duration comparison”, the time using electrocauterization is given first, and the time using ligature is given second. SD. * stands for the amount of the stars assessed by the Newcastle-Ottawa Scale. A hyphen is written where the scale is not applicable.

**Table 2:** Estimates of the random effects model (k = 8).

	Estimate	se	Z	*p*	CI Lower Bound	CI Upper Bound
Intercept	−0.3474	0.293	−1.28	0.202	−0.949	0.201

Abbreviations: se = standard error; CI = confidence interval.

**Table 3:** Random effects model (k = 3).

	Estimate	se	Z	*p*	CI Lower Bound	CI Upper Bound
Intercept	−7.28	3.07	−2.37	0.018	−13.304	−1.254

Abbreviations: se = standard error; CI = confidence interval.

## Data Availability

The datasets generated and/or analyzed during the current study are available from the corresponding author upon request.
